# Temperature Measurement and Numerical Prediction in Machining Inconel 718

**DOI:** 10.3390/s17071531

**Published:** 2017-06-30

**Authors:** José Díaz-Álvarez, Alberto Tapetado, Carmen Vázquez, Henar Miguélez

**Affiliations:** 1 Department of Aerospace Engineering, University Carlos III of Madrid, 28911 Leganés, Spain; 2 Department of Electronics Technology, University Carlos III of Madrid, 28911 Leganés, Spain; atapetado@ing.uc3m.es (A.T.); cvazquez@ing.uc3m.es (C.V.); 3 Department of Mechanical Engineering, University Carlos III of Madrid, 28911 Leganés, Spain; mhmiguel@ing.uc3m.es

**Keywords:** temperature sensor, fiber-optic, pyrometer, Inconel 718, machining

## Abstract

Thermal issues are critical when machining Ni-based superalloy components designed for high temperature applications. The low thermal conductivity and extreme strain hardening of this family of materials results in elevated temperatures around the cutting area. This elevated temperature could lead to machining-induced damage such as phase changes and residual stresses, resulting in reduced service life of the component. Measurement of temperature during machining is crucial in order to control the cutting process, avoiding workpiece damage. On the other hand, the development of predictive tools based on numerical models helps in the definition of machining processes and the obtainment of difficult to measure parameters such as the penetration of the heated layer. However, the validation of numerical models strongly depends on the accurate measurement of physical parameters such as temperature, ensuring the calibration of the model. This paper focuses on the measurement and prediction of temperature during the machining of Ni-based superalloys. The temperature sensor was based on a fiber-optic two-color pyrometer developed for localized temperature measurements in turning of Inconel 718. The sensor is capable of measuring temperature in the range of 250 to 1200 °C. Temperature evolution is recorded in a lathe at different feed rates and cutting speeds. Measurements were used to calibrate a simplified numerical model for prediction of temperature fields during turning.

## 1. Introduction

Ni alloys are commonly used in aircraft and power-generation turbines, rocket engines, and other aggressive environments due to their excellent mechanical properties combining high temperature strength, toughness, and corrosion resistance even at elevated temperatures, reaching operating temperatures close to 1000 °C [1].

These superalloys are classified as difficult-to-cut because of the elevated cutting forces, tool wear rate and high temperature involved during cutting, as a consequence of their mechanical properties and low thermal conductivity [2].

Cutting temperature strongly affects tool wear and is also a key parameter governing undesirable effects occurring at the workpiece, such as microstructural changes or tensile residual stresses [3,4]. For instance, the most used Ni alloy, Inconel 718, exhibits a face-centered cubic (FCC) structure γ matrix where the remaining phases reside. After a long thermal exposure at temperatures higher than 650 °C, the thermodynamically metastable phase γ’’ can be transformed into the stable phase δ (Ni3Nb), leading to a decreased performance of Inconel 718 [2].

Although traditional approaches to machining of thermoresistant alloys involve the use of cutting fluids [5], nowadays the most desirable technique is dry machining [6]. The use of traditional cutting fluid generates significant environmental and health problems for workers who come into contact with these substances [7,8]. Alternatives to the application of cutting fluid are studied by other authors, pointing out the high temperatures reached in the processes [9,10].

The main problem when machining low conductivity materials is the heat flowing to the workpiece due to the material removal process remaining in the freshly machined surface for a long time [11]. On the other hand, the elevated temperature reached in the workpiece leads to thermal softening of the material, decreasing the forces required for chip removal [4].

Measurement and control of temperature is still a challenge in the machining of thermoresistant alloys. It is worth noting the importance of controlling temperature for industry because of the risk of workpiece rejection (for instance, excessive residual stresses causing component distortion), enhanced tool wear [12] or microstructural changes further leading to a decrease in future component service life.

The difficulty of temperature measurement during machining is well known, being the focus of different works in the literature. Different experimental approaches have been developed, and are briefly summarized in the following paragraphs.

For instance, the use of a machinable thermocouple embedded in the workpiece for temperature measurement during milling of Inconel 718 was presented in [13], allowing the successive passes of the tool. A thermocouple combined with an infrared sensor has been used in [14], with the aim of measuring the tool–chip interface temperature when machining AISI 4140 alloy, obtaining values of maximum temperature close to the cutting zone around 525 °C. The attachment of a thermocouple close to the cutting zone is commonly a problem. Kus. et al. [14] solved this problem by developing a special apparatus for the thermocouple connection to the tool holder. Temperature measurement in Waste-to-Energy boilers using suction pyrometers, or high velocity thermocouples with low measurement uncertainty are described in [15], but they are not applied in measuring turning sections.

Infrared thermography has also been used in [16], to obtain temperature distributions at the chip–tool interface. This technique provided high accuracy for the target temperature range above 1250 K.

Tool temperature during turning and milling was measured in [17], using an infrared radiation pyrometer. It was found that this pyrometer allowed the measurement of the temperature history of a Cubic Boron Nitride (CBN) tip at the rake face between up-cutting and down-cutting.

Temperature measurement in drilling, being a critical process due to the difficult cooling, was carried out with a two-color pyrometer [18].

A strong effort in measuring temperatures during cutting has been carried out, however problems remain unsolved since most authors focusing on experiments report difficulties. On the other hand, although numerical modeling has been widely used to model metal cutting, mainly focusing on mechanical aspects of the process, this technique has been poorly used for simulating thermal issues. Main problems relate to the difficulty of combining chip removal simulation, in general requiring an explicit integration approach [19], and the need to simulate long cutting time in order to ensure steady state conditions, leading to extremely large calculation time.

Previous work of the authors has focused on simulation of the machining of Inconel 718, analyzing the effect of strain hardening and thermal softening during cutting [3]. The computational cost of these simulations was very elevated, even for the simulation of the first tool pass.

In this paper, a simplified model based on a finite element (FE) approach for simulation of steady temperature distribution in turning Inconel 718 is presented. The model is calibrated with precise temperature measurements made using a special purpose device, based on a two-color fiber-optic pyrometer [20,21,22]. The aim of this paper is to provide a measurement method for temperature control during the process and to provide information for the calibration of the numerical model suitable for prediction of thermal issues in Inconel cutting.

## 2. Experimental Setup

### 2.1. Lathe Setup and Instumentation

**Figure 1:**
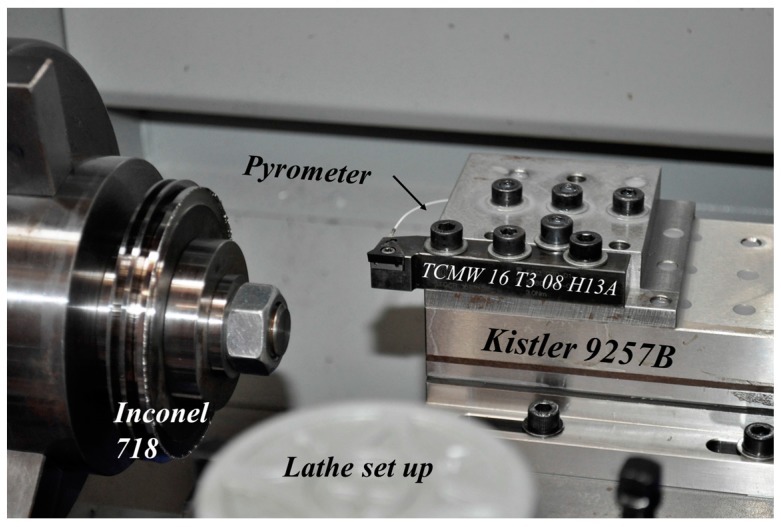
Test setup.

Orthogonal cutting tests were carried out in a Pinacho Smart lathe 6/165 (Huesca, Spain). The lathe was instrumented with a dynamometer model Kistler 9257B (Winterthur, Switzerland) with the aim of measuring cutting forces, and giving valuable information about the cutting process. The pyrometer was implemented inside the tool holder in order to measure the temperatures reached at the machined surfaces during the process (more details are presented in the subsequent section). The workpiece was a disc with a diameter of 100 mm and thickness of 2 mm, made of Inconel 718 (see the experimental setup in Figure 1).

The selected cutting tool was a triangular uncoated carbide insert (TCMW 16 T3 08 H13A, Sandviken, Sweden) with rake angle 0°, clearance angle 7° and cutting-edge radius 25 μm. The performance of the tool in terms of cutting force and temperature stability during the test was adequate.

The tests were carried out in dry conditions. The cutting parameters used in the experiments are summarized in Table 1.

### 2.2. Pyrometer Design and Calibration Setup

**Figure 2:**
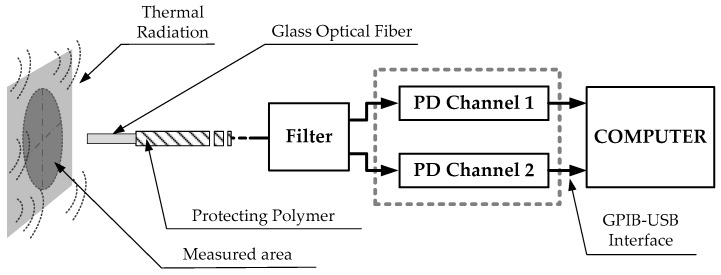
Schematic of the experimental setup.

**Figure 3:**
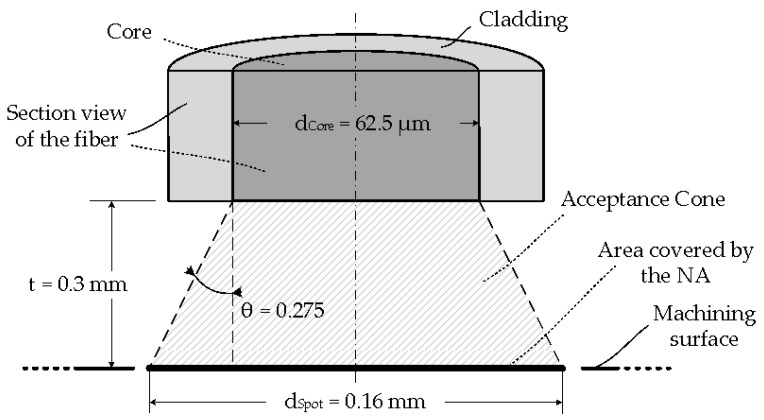
Schematic of the fiber probe on the measurement area.

Figure 2 shows the scheme of the setup used to measure the work piece temperature [20,21,22]. The temperature sensor is made of standard glass optical fiber with core (d_Core_) and cladding (d_Cladding_) diameters of 62.5 and 125 μm, respectively. This fiber allows the designed sensor to measure highly localized temperature measurements without using focusing lenses. Using a fiber numerical aperture (θ) of 0.275 and taking a distance (t) between the fiber end and the machining surface of 0.3 mm, the measure spot diameter (d_Spot_) is 0.16 mm, as depicted in Figure 3. The system is designed to be able to split the radiation collected by the optical fiber into two spectral bands centered at 1.3 and 1.55 μm, respectively. A dual InGaAs photodetector with constant responsivity values at both spectral bands is used to convert the light power to an electrical signal.

**Figure 4:**
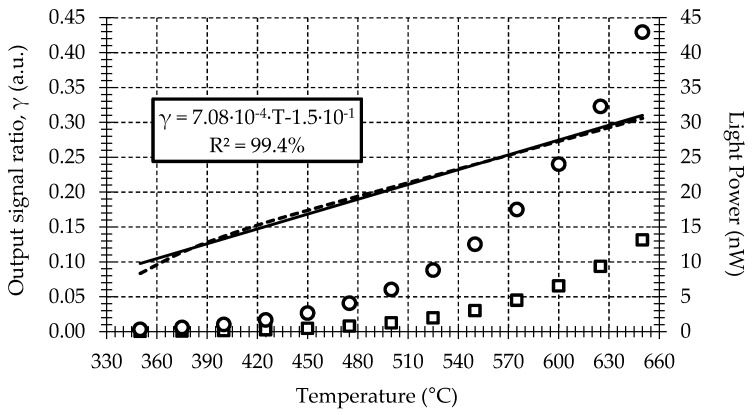
Calibration curve for: (□) 1.3 μm; (○) 1.55 μm; (–) 1.3/1.55 μm.

The two-color pyrometer is calibrated using a dry block calibrator and a blackbody kit. The calibration system allows a maximum temperature uncertainty of ±0.17 °C in the range of 50 to 650 °C. To perform the calibration curve, the fiber sensor is fixed at 0.3 mm from the blackbody using a calibrated metallic holder. The radiant flux emitted by the blackbody has been characterized at 1.3 and 1.55 μm spectral bands, while the temperature of the blackbody surface changes from 350 to 650 °C at 25 °C intervals. Measurements for this calibration are shown in Figure 4. The sample rate and number of samples at each temperature and wavelength are fixed at 1 kHz and 10 S, respectively. To ensure the stability of the measurements, a time interval of 45 min between each temperature measurement is taken during the calibration procedure. Finally, using computer software, the average of the measured optical power ratio at both spectral bands for each temperature is calculated from the mean of the samples.

The ratio between the measured power at 1.3 and 1.55 μm for each temperature is then calculated. This result is also shown in Figure 4. The sensitivity obtained is 7.08 × 10^−4^ °C^−1^. A statistical analysis of the ratio calibration curve shows a full-scale temperature error and lineal regression coefficient of 5% and 99.4%, respectively.

### 2.3. Pyrometer Validation

**Figure 5:**
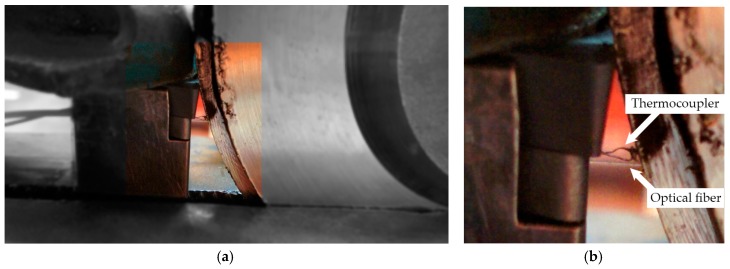
(**a**) Photograph of the fiber-optic pyrometer and the thermocouple on the machining surface. Inset included of the highlighted area; (**b**) detailed photograph of the pyrometer and thermocouple.

An experimental setup with a second temperature sensor for validation is realized to check the accuracy of the temperature measurements provided by the pyrometer on the machining surface. The procedure is carried out using the same mechanical setup used in a turning process, but without feed motion applied on the lathe. A workpiece made of heat-treatable Inconel 718 disk is located on the mandrel. To simulate the heat transferred by the cutting process to the workpiece, a heat gun is used. The fiber probe is placed into the tool insert following the procedure described in the preceding section. In addition to the fiber probe, a K-type thermocouple with a diameter of 0.13 mm is also introduced into the hole of the tool insert. The thermocouple junction is placed on the machined surface and, very close to the viewing area of the fiber probe, in order to ensure that both sensors are measuring on the same area; see Figure 5. The temperature is measured synchronously by the thermocouple and the pyrometer with a sample rate and number of samples of 1 Hz and 10 S, respectively.

**Figure 6:**
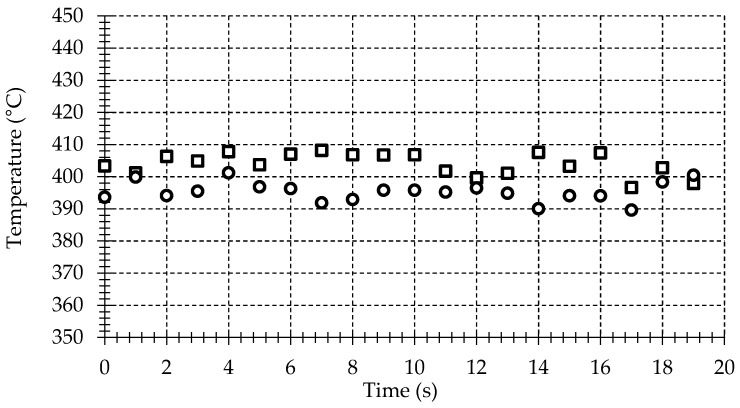
Temperature measurements for: (□) fiber-optic pyrometer; (○) K-type thermocouple.

The results depicted in Figure 6 show that the measured thermocouple temperature is quite lower than that showed by the pyrometer. This difference is due to the thermal contact resistance between the metal surface and the thermocouple junction, the thermocouple response and the temperature gradient to the surrounding.

Temperature measurements show similar values between the fiber-optic pyrometer and the thermocouple. The standard temperature error for a K-type thermocouple is 1.1 °C or 0.4% above 0 °C. Smaller uncertainties could be provided using high velocity thermocouples [15], but this is out of the scope of this work. A more detailed study of different variables affecting the fiber-optic pyrometer response and the related errors can be found in the authors’ previous works [22].

It is important to point out the test in this section using the thermocouple is only to verify the pyrometer calibration curve. The thermocouple is not useful for measuring surface temperature in material removal process such as turning. The measurements in the lathe require non-contact sensors for avoiding severe damage on them. A fiber-optic pyrometer will be used instead in the validation of the numerical modelling.

## 3. Numerical Model

As was discussed in the introduction, numerical modeling including simulation of chip removal could be an unaffordable way to address the study of the temperature reached at the freshly machined surfaces generated during workpiece machining. The computational cost is extremely high since the simulation of steady state conditions is required. Moreover, part of the heat that goes to the workpiece comes from the friction between the tool flank surface and the newly created surface, meaning that element erosion, required in order to avoid excessive distortion, makes the simulation of this frictional heating at the tertiary cutting zone difficult.

In order to have a more realistic prediction of the temperature field resulting from the chip removal process, a simplified thermal model was developed using the commercial finite element code ABAQUS.

**Figure 7:**
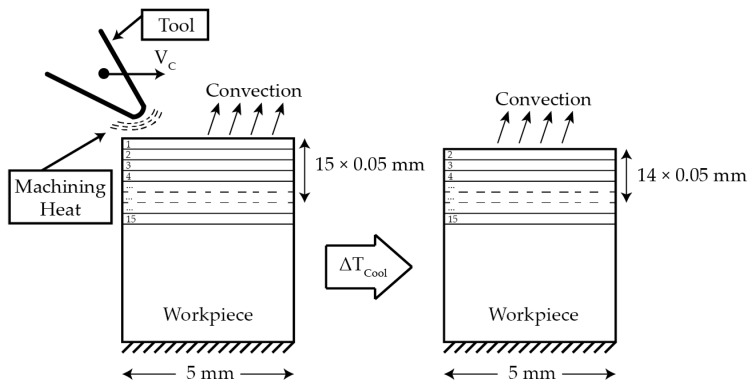
Model boundary condition for the model with a feed of 0.05 mm.

The thermal model is based on a heat source, modelled as a Gaussian function, moving at the cutting speed and applying an amount of heat on the machined surface equivalent to that caused by the action of the cutting tool during machining. A dflux subroutine is used to implement the heat source in the model. The amount of heat transmitted to the workpiece is calculated in terms of cutting conditions. The temperature obtained from the experiments is used for calibrating the amount of heat transmitted to the work piece. In order to take into account the effect of subsequent passes occurring in real turning operations, an iterative process is implemented in the model. After each pass of the heat source and during the time equivalent to one revolution, a thin layer with a thickness equal to the feed is removed from the model, then the heat source was applied to the next layer; see the scheme in Figure 7.

The model is meshed using approximately 50,000 CPE4T; the chosen element is a coupled temperature-displacement plane strain element, with 4 nodes, and bilinear displacement and temperature in each of them. Elements in the case of the maximum feed value were considered (cases with a feed = 0.15 mm/rev). A convection flux coefficient of 22.6 W/m^2^ was considered on the top surface [23]. A sink temperature of 293 K (measured mean room temperature) was considered on the bottom surface. The properties of Inconel 718 are summarized in Table 2, Table 3 and Table 4 [3].

## 4. Experimental and Numerical Results

### 4.1. Experimental Results

**Figure 8:**
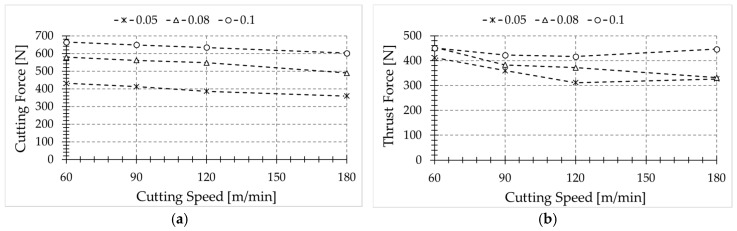
Cutting force (**a**) and thrust force (**b**) obtained for different cutting conditions.

Cutting forces were obtained from cutting tests for each case (see Figure 8). The experimental data (cutting forces and temperature level at the freshly machined surface) were used in order to estimate the energy involved in the turning process (see Equation (1)) and the heat partition.

**Figure 9:**
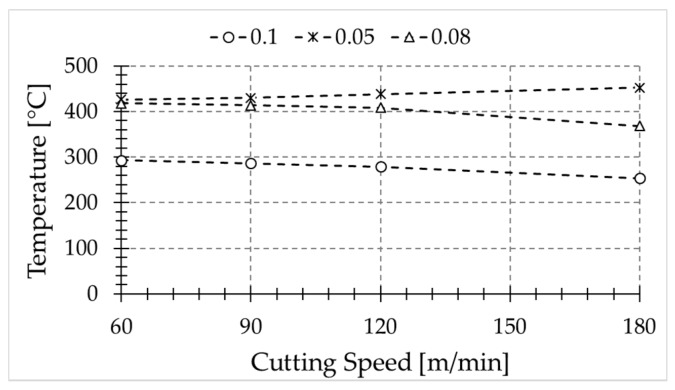
Temperature evolution of the new machined surface as a function of the feed rate: (*) 0.05 mm; (△) 0.08 mm; (○) 0.1 mm.

The temperatures at the freshly machined surface were recorded from each test; Figure 9 shows the temperature in the sixth pass. The workpiece temperature increases per revolution, thus the represented temperature should correspond to a specific number of passes.

Temperature slightly decreases with cutting speed, for feed rates equal to 0.08 and 0.1 mm. However, the opposite trend is observed for the lowest feed value (0.05 mm). It is worth noting that the cutting-edge radius is very close to this feed rate value, thus the effective rake angle is highly negative, resulting in larger deformation and increased temperature at the machined surface.

### 4.2. Numerical Results

**Figure 10:**
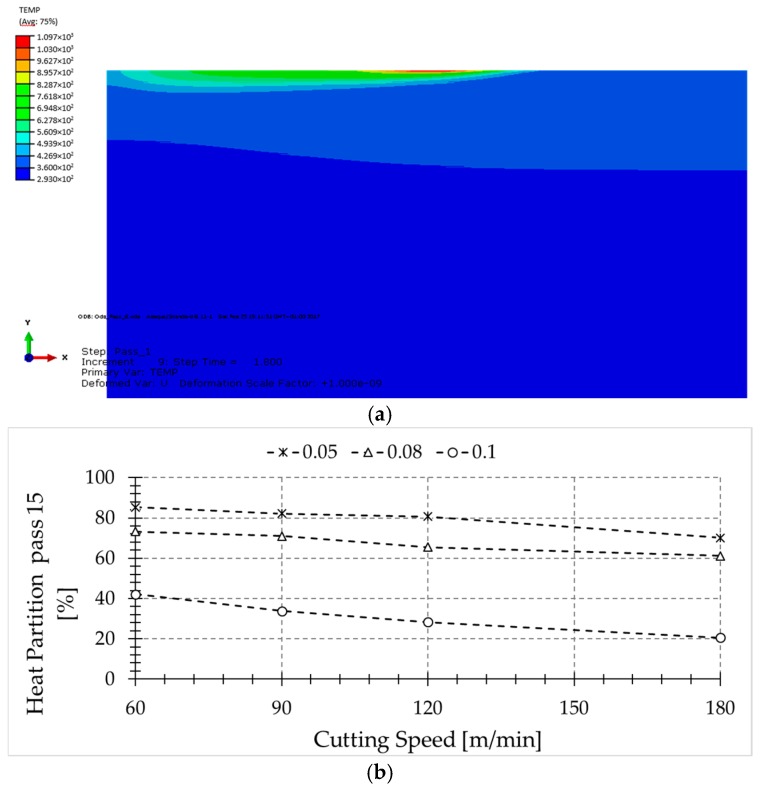
(**a**) Temperature field in the sixth pass under the following cutting condition: V_c_ = 60 m/min, f = 0.1 mm/rev; (**b**) Equivalent heat partition in function of different cutting parameters.

As explained previously, the information extracted from the experiments was used to calibrate a 2D thermal model (see Figure 10a). The model consists of a moving heat source which heats up the workpiece, simulating several passes. The heat source is calibrated with the experiments, obtaining the heat flux to the workpiece. In Figure 10b, it is estimated that the percent of heat that flows to the workpiece as a function of the cutting power (denoted heat partition) in pass number 15 is:

P_c_ = V_c_F_c_ (1)

where P_c_ is the cutting power, V_c_ is the cutting speed and F_c_ is the cutting force.

From Figure 10b, it can be deduced that for the larger feed rate values, the heat partition is much lower than for the lowest feed. Hence, from the point of view of the material removal rate, the process with the highest feed rate and speed is the best. Concerning heat partition, lower feed rate values, in the order of cutting-edge radius, lead to an enhanced level of heat being transmitted to the workpiece due to the aggressive conditions of the cutting, involving high deformation of the workpiece material.

In the literature, the given values of heat flowing to the workpiece are, in general, lower than the values obtained in this paper, due to the differences in cutting conditions. Typically, larger feeds are used in experiments, which means that the heat flow must cover larger distances to enter into the workpiece, and higher velocities, which implies less time to transmit the generated heat to the workpiece. The authors reported around 17% of the primary heat zone flowing into the workpiece. However, higher values, in the order of 50%, are found for low metal removal rates [24]. Furthermore, most of the analytical models used for temperature prediction during the machining do not take the cutting-edge radius into account, which plays an important role in the heat created in the primary zone and finally goes to the piece. This effect is more severe for small feed values, such as the value of 0.05 mm (in the order of cutting-edge radius) where the heat generated because of the rounded tip of the tool plays an important role [25].

**Figure 11:**
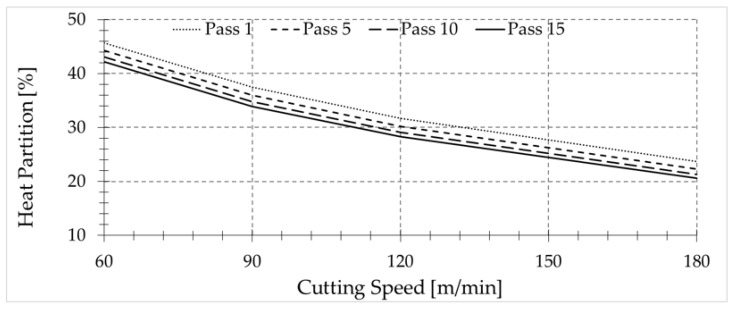
Heat partition in function of the number of passes for a feed rate equal to 0.1 mm/rev.

In addition, the way in which authors of various works obtain the amount of heat that flows to the workpiece makes it difficult to know the exact amount of heat that really goes into the workpiece after a single pass, since part of the heat that goes to the piece is removed by the chip in subsequent passes [26,27]. In Figure 11, the dependence of the percentage of heat transmitted to the workpiece on the number of passes is shown.

The trends found for the amount of heat entering the workpiece for small feed are in agreement with experimental studies conducted by other authors in grinding processes, where the cutting speeds are larger and thus the chip removal mechanisms can be assumed to be similar to that occurring in machining with a highly negative rake angle. In these experiments, most of the generated heat, in the order of 80% of the total power, was conducted into the workpiece [28,29,30].

**Figure 12:**
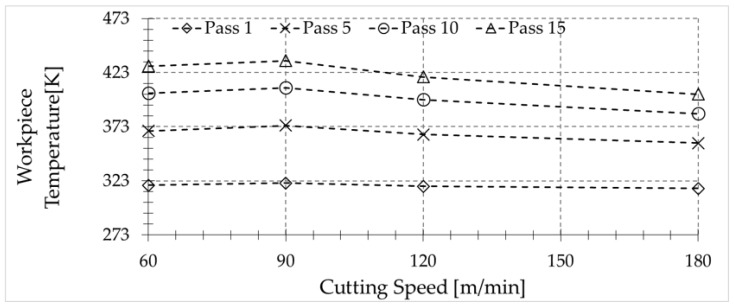
Temperature reached by the workpiece for a feed rate of 0.1 mm/rev after 1–15 passes for the different cutting speeds.

Taking the previous discussion into account, it is worth showing the variation of the temperature at the surface of the workpiece just before being removed by the chip after each pass (see Figure 12). The level of temperature depends on the geometry of the workpiece and the cutting parameters which determine the amount of heat transferred to the workpiece, and the characteristic time of the heat dissipation in the workpiece. However, for the tested conditions, it is clear that the workpiece is unable to dissipate the amount of heat generated during the machining, due to its low thermal conductivity.

## 5. Conclusions

This paper focuses on the measurement and prediction of temperature during turning of Inconel 718.

The temperature sensor is based on a pyrometer used for temperature measurement, and has demonstrated good performance in measuring temperature in hard environments where emissivity can play an important role. Considering the huge gradient of temperatures in the measured area, the pyrometer is capable of recording more accurate measurements than those obtained using other techniques. Since the spot diameter of the pyrometer is 0.16 mm, the measured values are the average temperature of the area covered by the spot.

The measured temperatures showed that, for the range of cutting speeds considered in this paper, when the cutting speed increases, the temperatures reached in the newly machined surface slightly decrease for large feed values. In addition, the increment of the feed leads to a decrement in the recorded temperatures in the analyzed range, probably due to the longer time required to propagate the heat across the uncut chip thickness removed in the subsequent pass.

For low feed values close to the cutting-edge radius of the tool, it was observed that the highest level of temperature occurs at the machined surface. Hence, the risk of workpiece damage due to change of microstructure is enhanced in these conditions.

A simplified thermal model was developed with the aim of predicting the temperature achieved in the machining process. The model was calibrated with the experimental data obtained from the tests. Using this model, the amount of heat transmitted to the workpiece for all cases analyzed was obtained. The numerical model allowed the effect of multiples passes in the temperature reached by the workpiece to be taken into account. The methodology presented in this paper can be extrapolated to a non-orthogonal cutting process. On the other hand, the model can simulate different cooling times, different methods to cool down the piece and its effects on the reached temperatures.

## Figures and Tables

**Table 1:** Cutting parameters in turning tests.

Depth of Pass (mm)	Feed Rate (mm/rev)	Cutting Speed (m/min)
2	0.05, 0.08 and 0.1	30
		60
		90
		120
		180

**Table 2:** Temperature dependence of thermal conductivity for Inconel 718.

Thermal Conductivity (W/m/K)	Temperature (K)
10.31	293
11.88	373
13.6	473
16.6	673
20.1	873
26.3	1073
30.75	1573

**Table 3:** Temperature dependence of specific heat capacity for Inconel 718.

Specific Heat Capacity (J/kg·K)	Temperature (K)
362	293
378	373
400	473
412	673
460	873
1073	1073
1573	1573

**Table 4:** Inconel 718 physical properties.

Density (kg/m^3^)	Poisson Ratio	Modulus of Elasticity—293 K—(Gpa)
8300	0.3	217
